# Case report: MOG-IgG-associated encephalitis with Epstein-Barr virus infection and Alzheimer's pathologic change in cerebrospinal fluid

**DOI:** 10.3389/fneur.2022.1013413

**Published:** 2022-12-02

**Authors:** Lin Li, Chuan Li, Dan Yao, Yun-feng Hao, Chao Zhao, Qi Yan, Jun-tong Liu, Shu-yu Liu, Wen-ping Zhu, Ying Du, Wei Zhang

**Affiliations:** ^1^Department of Neurology, Tangdu Hospital, Fourth Military Medical University, Xi'an, China; ^2^Xi'an Medical University, Xi'an, China

**Keywords:** MOG-IgG associated encephalitis, Epstein-Barr virus, cerebrospinal fluid biomarkers, Alzheimer's disease, rituximab

## Abstract

Immunoglobulin G antibodies to myelin oligodendrocyte glycoprotein (MOG-IgG) associated disease is a rare, demyelinated disease in the central nerve system (CNS) predominately involving optic nerve, spinal cord, and brain leading to optic neuritis (ON), transverse myelitis (TM), encephalitis. The phenotype of MOG-IgG-associated encephalitis is similar to acute disseminated encephalomyelitis (ADEM) presenting with seizures, abnormal behavioral and psychological symptoms, and cognitive impairment. A few brain biopsies show multiple sclerosis (MS) pattern histopathology with T cells, macrophages, and complement activation. To date, how MOG-IgG is produced is unknown. Herein, we report a case of a 32-year-old male with MOG-IgG-associated encephalitis presenting MOG-IgG in cerebrospinal fluid (CSF) but seronegative, as well as Epstein-Barr virus (EBV) infection and Alzheimer's pathologic change in CSF (Aβ42 = 317 pg/ml, T-Tau = 538 pg/ml, p-Tau =10.09 pg/ml). With a combination treatment of administering intravenous immunoglobulin (0.4 mg/kg/d, 5 days) with a low dose of methylprednisolone (80 mg/d, 5 days) and rituximab (100 mg/week, 3 weeks), the patient recovered significantly after 3 months follow-up. This case provides us with new thoughts into the production of MOG-IgG and the possible pathologic mechanism of MOG-IgG-associated disease (MOG-AD) and simultaneously further confirms the interaction between EBV and changes of CSF biomarkers of Alzheimer's disease (AD).

## Background

Myelin oligodendrocyte glycoprotein (MOG) is a myelin glycoprotein specifically expressed on oligodendrocytes of the central nervous system (CNS). Numerous studies have established a pathogenic role of MOG-IgG antibodies in the animal model of experimental autoimmune encephalomyelitis (EAE) (1). At present, it is believed that MOG-IgG antibody mediates a unique group of acquired demyelinating syndromes, delineating a disease with acute disseminated encephalomyelitis (ADEM), optic neuritis (ON) or transverse myelitis (TM), termed MOG-Ab associated disease (MOGAD) (2). MOGAD is different from multiple sclerosis (MS) and aquaporin-4 (AQP4)-positive neuromyelitis spectrum disease (NMOSD) (3). MOG-IgG antibodies in serum are pathogenetic and could cross the blood-brain barrier (BBB) in an inflammatory environment. Tissue injury can occur *via* antibody-mediated injury or MOG-reactive T cell-induced inflammation. However, the mechanisms leading to the selective production of MOG-IgG antibodies and BBB dysfunction are unclear.

Attacks can be preceded by virus infections which have been proposed as potential triggers of MOGAD (4, 5). Nakamura reported a case of a patient who developed MOG-IgG antibody-positive ADEM following infectious mononucleosis (IM) due to Epstein–Barr virus (EBV) infection (6). The presence of IgM and IgG of EBV viral capsid antigen in serum, and the absence of EBV genome in cerebrospinal fluid (CSF) samples is strong evidence for an autoimmune pathogenesis of neurological signs following IM. However, no MOG-IgG antibody-positive ADEM cases with a preceding EBV infection in CSF have been reported to date.

Herein, we report a case of a 32-year-old male diagnosed with MOG-IgG-associated encephalitis presenting with MOG-IgG in CSF but seronegative, as well as EBV infection and Alzheimer's pathologic change in CSF. We aim to supply new insights into the production of MOG-IgG and the possible pathologic mechanism of MOG-AD and confirm the interaction between EBV and changes in CSF biomarkers of Alzheimer's disease (AD).

## Case presentation

A 32-year-old man was hospitalized in our hospital complaining of headaches and fever for more than 1 month and speech disorders for more than half a month. More than 1 month before admission, the patient had a paroxysmal needle-pricking headache accompanied by a low fever, the temperature measured at 37.4°C. The patient was treated with indomethacin and pregabalin after the normal plain computer tomography (CT) scan of the head. After taking the medicine for more than 10 days, his headache and fever symptoms improved, but the patient felt that his tongue was stiff, his speech was slow, and not fluent. His speech difficulties got worse in time. He had difficulty finding words, retelling sentences, naming objects, and calculating numbers. He could only speak two or three words but understood what people said and there was no memory loss, seizures, or mental behavior abnormalities during the course of the disease. The patient had been healthy, and nobody had similar symptoms or related family history of autoimmune diseases and dementia in his family.

**Figure 1:**
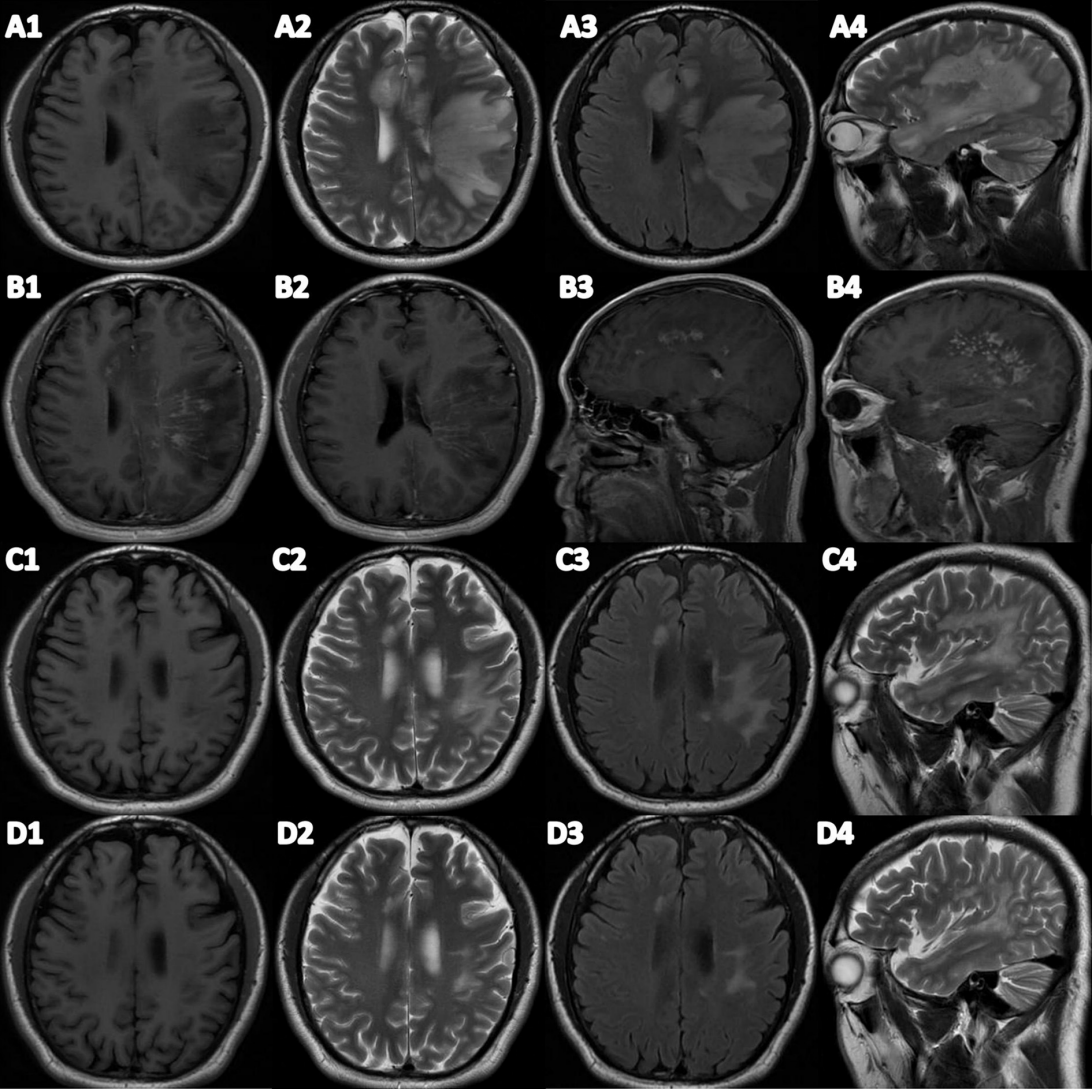
**(A1–A4)** Brain plain MRI before treatment. **(B1–B4)** Brain T1-enhanced MRI. **(C1–C4)** Brain plain MRI after treatment. **(D1–D4)** Brain plain MRI after 3 months of treatment. **(A1/C1/D1)** Transverse T1 weight imaging. **(A2/C2/D2)** Transverse T2 weight imaging. **(A3/C3/D3)** Fluid attenuated inversion recovery (FLAIR). **(A4/C4/D4)** Sagittal T2 weight imaging.

**Figure 2:**
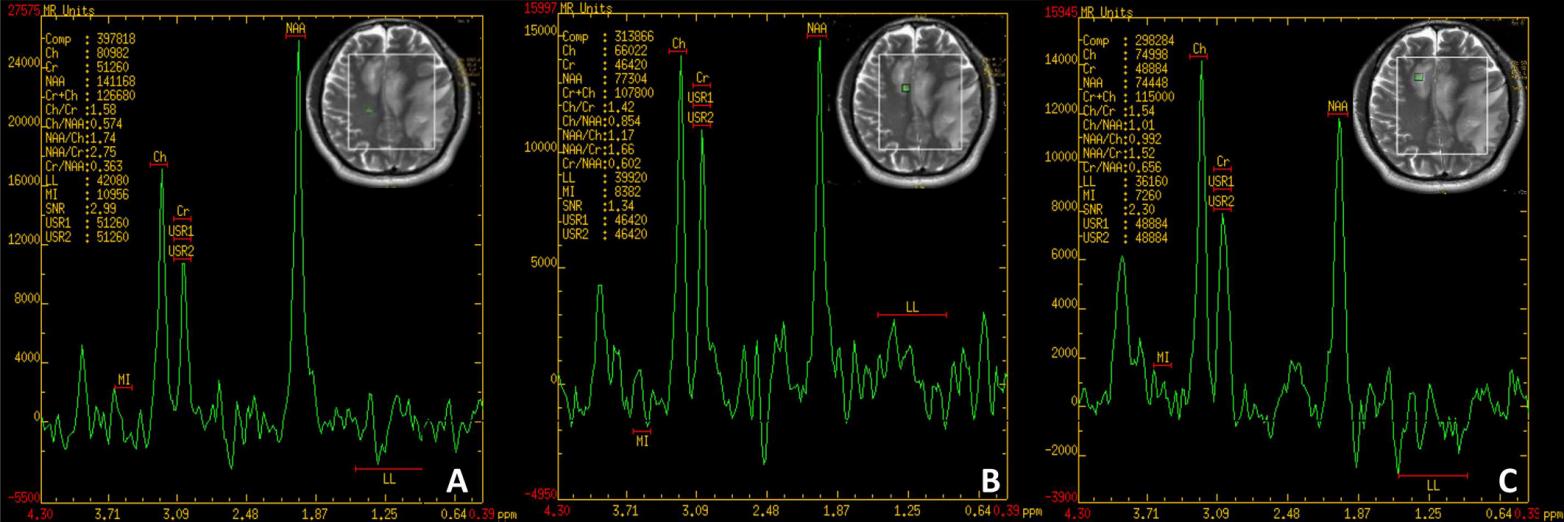
**(A)** Normal region. NAA/Cho = 1.74. **(B)** Edge of the lesion. NAA/Cho = 1.17. **(C)** Center of the lesion. NAA/Cho = 0.992. NAA, N-acetyl aspartic acid; Cho, Choline.

A physical examination found the patient's speech was non-fluent and his calculation ability was decreased. The Babinski sign and Chaddock's sign on the right side were positive. We made neuropsychological evaluations the day after admission in which the patient scored 20/30 on the Mini-Mental State Examination (MMSE) and 20/60 on the Boston Naming Test (BNT) with 16 years of education and graduating from college. Cranial magnetic resonance imaging (MRI) plain scans revealed hypointensity on T1-weighted image, hyperintensity on T2-weighted image and fluid-attenuated inversion-recovery (FLAIR) signals in the bilateral semi-oval center, left frontotemporal parietal insula, right frontal lobe, left internal capsule hindlimb, left thalamus, left cerebral peduncle, and left pons (Figure 1). Enhanced cranial MRI showed linear and nodular enhancement within the lesions (Figure 1). Magnetic resonance spectroscopy (MRS) imaging showed that the N-acetyl aspartic acid (NAA) peak decreased significantly in the center and the edge of the lesions, and the choline peak was normal, suggesting demyelinating lesions (Figure 2). The positron emission tomography/computed tomography (PET/CT) scan of the whole body presented reduced glucose metabolism of cranial lesions (Supplementary Figure 1). Routine blood tests were normal. His CSF exhibited slightly increased white cell counts of 14 × 10^6^/L (normal range: 0–10 × 10^6^/L) and protein levels of 540.9 mg/L (normal range: 80–430 mg/L) with normal glucose and chlorine levels. Exfoliative cytology of CSF showed the proportion of lymphocytes accounted for 78% and monocytes 22%. According to the flow cytometry immunophenotype analysis of blood, CD19+ B cells accounted for 27.89% of total lymphocytes and CD20+ B cells 22.05%. EBV was discovered by next-generation sequencing (NGS). MOG-IgG was detected in CSF (titer: 1:10) but seronegative using a cell-based assay (CBA). Total tau (T-tau) was measured to evaluate the neuronal injury. Then AD biomarkers in CSF were present as Aβ42 317 pg/ml, T-Tau 538 pg/ml, and p-Tau 10.09 pg/ml (Table 1). Autoimmune encephalitis and paraneoplastic antibodies were both negative in serum and CSF (Supplementary Tables 1, 2). Quantitative analysis of serum and CSF protein indicated that the albumin in both was within the normal reference range and the values of IgG, IgA, and IgM (68.6, 8.36, 3.29 mg/l) were all elevated in CSF but normal in serum revealing slightly impaired BBB function. IgG-oligoclonal bands (OB) are only seen in CSF but not in serum representing central intrathecal synthesis by isoelectric focusing immunofixation. The levels of folic acid, vitamin B12, homocysteine, thyroid hormone, and related antibodies and tumorous markers were in the normal range. Other laboratory tests, such as the spectrum of antinuclear antibodies, human immunodeficiency virus (HIV) antibodies, and syphilis antibodies, were negative.

**Table 1:** Test results of CSF biomarkers of AD.

	**First results**	**Second results**	**Third results**	**Reference interval (pg/ml)**
Aβ42	317	373	983	610–974
p-Tau	10.09	21.33	29.14	19.66–45.67
T-Tau	538	>2,000	118	47–225

CSF, cerebrospinal fluid; AD, Alzheimer's disease; Aβ, amyloid β-protein; p-Tau, phosphorylated-Tau; T-Tau, total-Tau.

Because of the virus infection, we administered intravenous ganciclovir antiviral therapy 0.25 g twice a day for 10 days in the beginning. On the fourth day after admission, we received the reports of NGS and antibodies, and MOG-IgG-associated encephalitis was diagnosed. We gave immunoglobulin intravenously at 0.4 g/kg/d per day for 5 days and further completed PET/CT examination due to the space-occupying effect of intracranial lesions. PET/CT showed no solid tumors. Methylprednisolone was administered intravenously at 80 mg per day for 5 days, and then was taken orally at 32 mg per day with a weekly reduction of 8 mg. Rituximab was administered intravenously at 100 mg once a week for 3 weeks to reduce autoimmune B cells. After the third rituximab treatment, the patient no longer suffered from the headache and his speech difficulties improved obviously. He could speak and retell sentences fluently, name objects correctly, and calculate numbers precisely. Neuropsychological examinations were reevaluated in which the patient scored 28/30 on MMSE, 58/60 on BNT, and 26/30 on the Montreal Cognitive Assessment (MoCA). Compared to the previous scans, MRI plain scans of the head showed that lesions of the right frontal insula, the left hind limb of the internal capsule, and the left thalamus had been absorbed and lesions of the bilateral semioval center and the left temporal parietal insula, the left cerebral foot, and the left pontine were smaller than the front (Figure 1). The white cell counts were 6 × 10^6^/L and protein levels of 215 mg/L in CSF were normal. EBV in serum and CSF were negative by NGS. The titer of MOG-IgG was reduced to 1:1 in CSF and was consistently negative in serum. CSF biomarkers were still presented with AD pathologic changes: Aβ42 373 pg/ml, T-Tau > 2,000 pg/ml, p-Tau 21.33 pg/ml) (Table 1).

During the follow-up, the patient achieved complete recovery in the third month. All tests of cognitive domains and language function were normal. Brain MRI scan only showed reduced abnormal signals in the left semioval center. No EBV was found in CSF with normal white cell and protein levels. But the titer of MOG-IgG in CSF remained at 1:10. CSF biomarkers revealed that AD pathologic changes returned to normal: Aβ42 983 pg/ml, T-Tau 118 pg/ml and p-Tau 29.14 pg/ml (Table 1).

## Discussion

Linington et al. first detected the MOG-IgG antibody in an animal model of EAE in 1984 and confirmed that the antibody was pathogenic and could aggravate the demyelination (7). It was found that the clinical phenotypes of patients with MOG-IgG positivity were mostly recurrent ON, ADEM, and AQP4-negative myelitis. These patients had specific characteristics in epidemiology, clinical manifestations, imaging changes, and response to treatment. MOG-AD was ultimately defined as an independent disease entity. In 2018, Jarius et al. published an international recommendation about the diagnostic criteria for MOG-AD which emphasized typical clinical phenotypes, MRI demyelination lesions in CNS, and seropositivity for MOG-IgG detected using a CBA employing full-length human MOG as target antigen (8).

For the patient, his clinical phenotype was ADEM, and cranial MRI showed demyelinated lesions with linear and nodular enhancements. Nevertheless, MOG-IgG was detected in CSF (titer: 1:10). But seronegative by CBA was defined as a “red flag” in the recommendation. To determine the diagnostic relevance of MOG-IgG in CSF of seronegative cases, a retrospective study was conducted in 2019 by analyzing the CSF of 80 MOG-IgG seronegative patients with demyelinating disease. It found that analyzing CSF could improve diagnostic sensitivity in seronegative patients and might provide novel insight into the biological mechanisms of the MOG antibody synthesis (9). The patient was ultimately diagnosed with MOG-IgG-associated encephalitis.

To date, the pathogenesis of MOG-AD and the mechanism of MOG-IgG production are unclear. Histopathology of a few brain biopsies showed demyelination within the lesions accompanied by complement deposition, macrophage activation, oligodendrocyte infiltration, T cells, and B cells infiltration in the perivascular and brain parenchyma (10). In animal models, it was found that the intestinal flora could assist the production and activation of MOG-specific CD4+ T cells and B cells promoting the production of MOG-IgG antibodies (11). Therefore, it is suggested that the pathological mechanism of MOG-AD is similar to MS, which is mediated by CD4+ T cells with B cells participation playing an important role (12). Due to the high MOG-IgG positive rate in serum, it was hypothesized that MOG antigen may leak into the periphery and be recognized by the immune system. When the permeability of BBB increased, such as the CNS infection, MOG-IgG entered the CNS causing demyelinated lesions. For our patient, we completed the NGS of the CSF finding EBV infection. EBV, which is also called Human Herpes Virus type 4 (HHV4), infects essentially all human beings at some time during their life span. The majority of infectious cases are occult infections that are related to lymphoma, nasopharyngeal carcinoma, and systemic autoimmune diseases like MS in later life (13). To our knowledge, EBV infection is related to the onset of MS, but there are currently very few reports about EBV and MOG-AD. Animal models have used hepatitis virus A59, Theiler's encephalomyelitis virus, Coxsackievirus B3, etc. to induce the production of MOG-IgG, suggesting that viral infection may induce the production of MOG-IgG (14). For the patient, the quotient (CSF/Serum) albumin value was raised slightly, suggesting slightly impaired BBB. In addition, IgG-oligoclonal bands (OB) are only seen in CSF by isoelectric focusing immunofixation, revealing synthesis in the central sheath. We consider that EBV infection causes mild BBB damage, and activated lymphocytes (including T cells, B cells, and plasma cells) infiltrate the brain. Then B cells act as antigen-presenting cells (APC) to present MOG antigen to T cells. Then anti-MOG antibodies are produced and cause demyelinated lesions by the activations of the complement system and the release of pro-inflammatory cytokines.

Accumulated evidence based on previous studies has shown that immune mechanisms and infectious agents played an important role in the pathogenesis of AD. EBV correlation with cognitive impairment has also been widely discussed (15–18). An 11-year follow-up study found that EBV infection was not significantly related to cognitive decline or dementia. Other studies and systemic reviews found that EBV infection increased the risk of AD. To explore the mechanism of EBV infection in the pathogenesis of AD, several cellular and animal experiments had been carried out (19, 20). The B cells of AD patients were immortalized by EBV to generate a lymphoblastoid cell line (LCL). It was found that the anti-Aβ antibody production of LCL of AD patients was significantly higher than the control group (21). In the mouse model, Aβ42 deposits were found in the brain after HSV1 infection (22). For the patient, we found that EBV infection and AD-like pathological changes coexisted, and the same manifestation was still observed after 1 month of treatment. At the same time, we found that CD20+ B cells in the patient's serum were significantly increased, further verifying that EBV infection activated B cells leading to the AD pathologic changes in CSF. Fortunately, AD pathologic changes in CSF disappeared after 3 months of follow-up. However, whether the AD pathological changes in CSF will appear again and whether or not the patient will show AD clinical phenotype in later life are not clear. Long-term follow-up is needed.

Corticosteroids, plasma exchange, and immunoglobulin were used as standard first-line treatments in the acute phase (23). With high-dose intravenous methylprednisolone, patients could usually get a recovery, but a few of them might have residual neurological deficits and MRI changes (24–27). A 2-month prednisone taper is recommended because of the risk of relapse with abrupt steroid discontinuation. But a UK study showed the risk of relapse was higher in those who were immunosuppressed for < 3 months (27). So long-term immunosuppressive therapy is necessary. A few cases used rituximab and mycophenolate mofetil to prevent recurrence (28). The persistence or disappearance of MOG-IgG could be related to the disease course and prognosis. For our patient, we treated him by combining immunoglobulin and methylprednisolone as first-line drugs. And low-dose rituximab was used as a long-term immunosuppressive drug. After 1 month of treatment, the patient's speech disorders improved significantly and almost returned to the normal level. The MOG-IgG titer in CSF decreased to 1:1. Cranial MRI showed that the intracranial lesions were absorbed significantly. After 3 months of treatment, the patient's clinical symptoms were fully recovered with only a few abnormal signals of the left semioval center. However, the MOG-IgG titer in CSF was back to 1:10 which was inconsistent with his clinical manifestations. According to past reports, MOG-IgG titers may fall below cut-off temporarily following treatment with steroids, plasma exchange, or immunosuppressants (or even spontaneously) and rise again at a later disease stage. Only some cases of monophasic MOG-positive ADEM in adult patients have been described in which MOG-IgG disappeared permanently following clinical recovery (29–31). Regular follow-up and evaluation are necessary.

## Conclusion

Altogether, we showed the clinical characteristics of a case of MOG-IgG-associated encephalitis with positive MOG-IgG in CSF. Simultaneously, we found that EBV infection coexisted with MOG-IgG-associated encephalitis revealing the relation between EBV infection and the production of MOG-IgG as well as the possible pathogenic mechanism of MOG-AD.

## Data availability statement

The original contributions presented in the study are included in the article/Supplementary material, further inquiries can be directed to the corresponding author/s.

## Ethics statement

The studies involving human participants were reviewed and approved by the Ethical Committee of Tangdu Hospital, Fourth Military Medical University. The patients/participants provided their written informed consent to participate in this study. Written informed consent was obtained from the individual(s) for the publication of any potentially identifiable images or data included in this article.

## Author contributions

YD and WZ had full access to all the data in the study, take full responsibility for the integrity of the data and the accuracy of the data analysis, and undertook the final manuscript review and editing. LL, CL, and DY drafted the manuscript. Y-fH, QY, and CZ carried out the data collection. J-tL, S-yL, and W-pZ carried out the patient follow-up. All authors had contributed to acquisition, analysis, interpretation of data, critical revision of the manuscript, and approved the submission.

## Funding

This work was supported by research grants from the National Natural Science Foundation of China (81971003, 82171406, and 82271457), Key Project of the Research and Development Plan of Shaanxi Province (2018SF-086), Key Project of the Basic Natural Science Research Program of Shaanxi Province (2019JZ-18), Tangdu Hospital Innovation Development Foundation (2018QYTS010, 2019QYTS002), Tangdu Hospital Clinical Research Project (2021LCYJ040), and Tangdu Hospital Foundation for Social Recruitment Talent (2021SHRC011).

## Conflict of interest

The authors declare that the research was conducted in the absence of any commercial or financial relationships that could be construed as a potential conflict of interest.

## Publisher's note

All claims expressed in this article are solely those of the authors and do not necessarily represent those of their affiliated organizations, or those of the publisher, the editors and the reviewers. Any product that may be evaluated in this article, or claim that may be made by its manufacturer, is not guaranteed or endorsed by the publisher.
