# Supplementary material for: Case report: MOG-IgG-associated encephalitis with Epstein-Barr virus infection and Alzheimer's pathologic change in cerebrospinal fluid
Source: Front Neurol. 2022 Dec 2;13:1013413. doi: 10.3389/fneur.2022.1013413 (PMC9755887; doi:10.3389/fneur.2022.1013413)
Supplement: Supplementary file 1 [file Table_1.doc]

Table 1 Autoimmune encephalitis antibodies

| Antibodies | Serum | CSF | Test method | Reference interval |
| --- | --- | --- | --- | --- |
| Anti-NMDAR IgG | - | - | CBA | - |
| Anti-AMPAR1 IgG | - | - | CBA | - |
| Anti-AMPAR2 IgG | - | - | CBA | - |
| Anti-LGI1 IgG | - | - | CBA | - |
| Anti-Caspr2 IgG | - | - | CBA | - |
| Anti-GABAR IgG | - | - | CBA | - |
| Anti-DPPX IgG | - | - | CBA | - |
| Anti-IgLON5 IgG | - | - | CBA | - |
| Anti-Glyα1 IgG | - | - | CBA | - |
| Anti-GABAARα1 IgG | - | - | CBA | - |
| Anti-GABAARβ3 IgG | - | - | CBA | - |
| Anti-GABAARγ2 IgG | - | - | CBA | - |
| Anti-mGluR5 IgG | - | - | CBA | - |
| Anti-D2R IgG | - | - | CBA | - |
| Anti-Neurexin3α IgG | - | - | CBA | - |

NMDAR:N-Methyl-D-Aspartate Receptor；-:Negative; CBA:Cell-Based Assay；

AMPAR:α-Amino-3-Hydroxy-5-Methyl-4-Isoxazole-Propionic Acid Receptor；

LGI1:Leucine-Rich Glioma Inactivated-1；Caspr2:Contactin Associated Protein-Like 2；

GABAR:[Gamma-Aminobutyric Acid](http://shortof.com/suolueci/Gamma_2DAminobutyric-Acid-GABA) Receptor; DPPX:Dipeptidyl-Peptidase-Like Protein 6; IgLON5:Immunoglobulin-Like Cell Adhesion Molecule 5; Gly:Glycine;

mGluR5:[Metabotropic Glutamate Receptor 5](https://www.sciencedirect.com/topics/neuroscience/metabotropic-glutamate-receptor-5#:~:text=Glutamate%20signaling%20through%20type%205%20metabotropic%20glutamate%20receptor,an%20important%20modulator%20of%20neuronal%20excitability%20and%20plasticity.); D2R: Dopamine 2 receptor.

Table2 Paraneoplastic antibodies

| Antibodies | Serum | CSF | Test method | Reference interval |
| --- | --- | --- | --- | --- |
| Anti-GAD65 IgG | - | - | immunospot method | - |
| Anti-Hu IgG | - | - | immunospot method | - |
| Anti-Yo IgG | - | - | immunospot method | - |
| Anti-Ri IgG | - | - | immunospot method | - |
| Anti-CV2 IgG | - | - | immunospot method | - |
| Anti-Ma2 IgG | - | - | immunospot method | - |
| Anti-Amphiphysin IgG | - | - | immunospot method | - |
| Anti-Ma1 IgG | - | - | immunospot method | - |
| Anti-SOX1 IgG | - | - | immunospot method | - |
| Anti-Tr(DNER) IgG | - | - | immunospot method | - |
| Anti-Zic4 IgG | - | - | immunospot method | - |
| Anti-PKCγ IgG | - | - | immunospot method | - |
| Anti-Recoverin IgG | - | - | immunospot method | - |
| Anti-Titin IgG | - | - | immunospot method | - |

GAD:Glutamate Decarboxylase; SOX: Sex Determining Region Y-Box; Zic: Zinc Finger Of The Cerebellum; PKC: Protein Kinase C;-: Negative
