# Supplementary figures and images for: Case report: MOG-IgG-associated encephalitis with Epstein-Barr virus infection and Alzheimer's pathologic change in cerebrospinal fluid
Source: Front Neurol. 2022 Dec 2;13:1013413. doi: 10.3389/fneur.2022.1013413 (PMC9755887; doi:10.3389/fneur.2022.1013413)

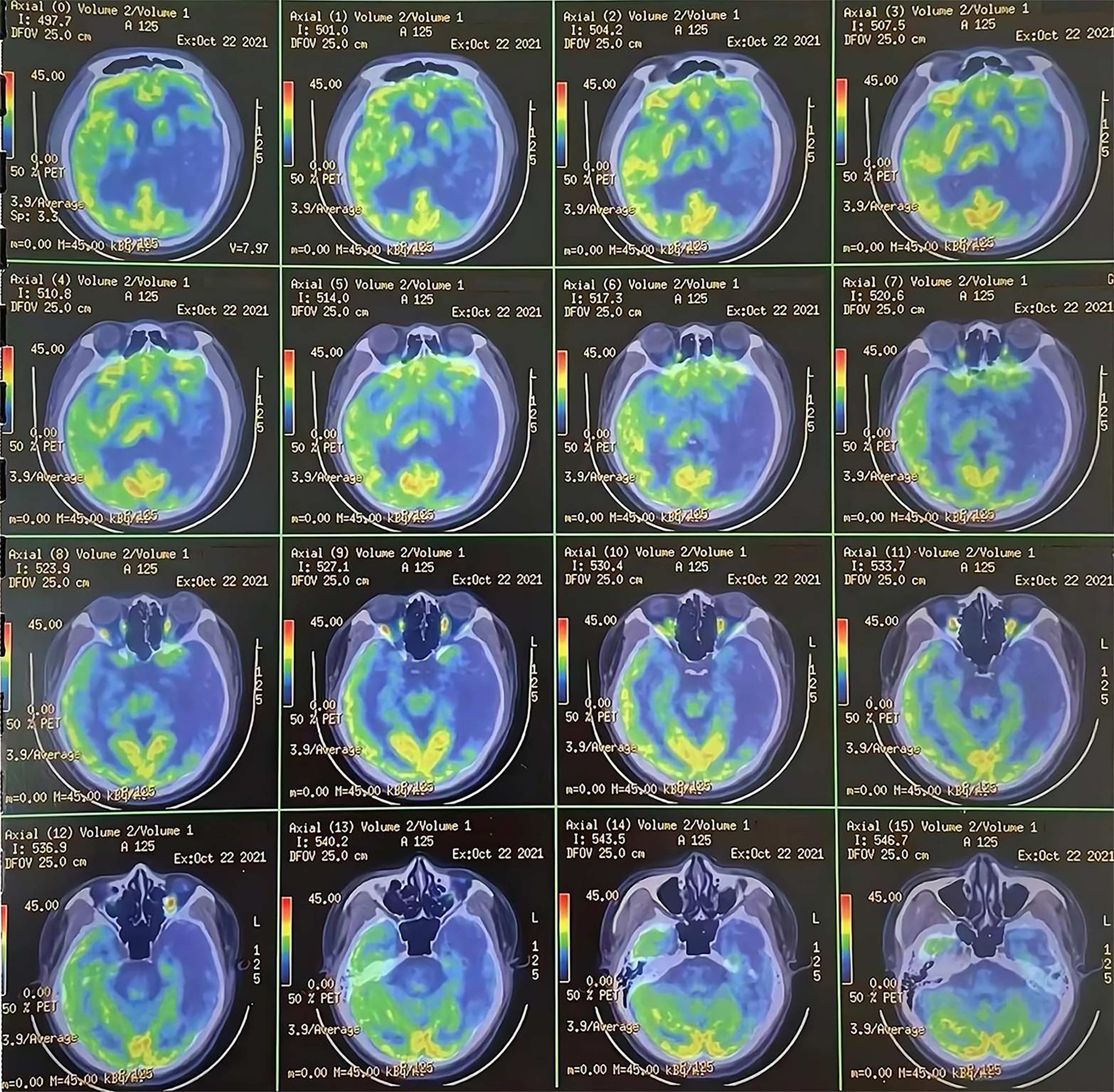

Supplement: Supplementary file 2 [file Image_1.jpeg]
